# Development and reliability of a scale of physical-activity related informal social control for parents of Chinese pre-schoolers

**DOI:** 10.1186/s12966-014-0087-y

**Published:** 2014-07-18

**Authors:** Yi-nam Suen, Ester Cerin, Robin R Mellecker

**Affiliations:** 1 Institute of Human Performance, The University of Hong Kong, Pokfulam Road, Hong Kong, SAR, People’s Republic of China; 2 Centre for Physical Activity and Nutrition Research, Deakin University, 221 Burwood Highway, Burwood 3125, VIC, Australia

**Keywords:** Nominal group technique, Health, Physical activity, Parental questionnaire, Reliability

## Abstract

**Background:**

Parents’ perceived informal social control, defined as the informal ways residents intervene to create a safe and orderly neighbourhood environment, may influence young children’s physical activity (PA) in the neighbourhood. This study aimed to develop and test the reliability of a scale of PA-related informal social control relevant to Chinese parents/caregivers of pre-schoolers (children aged 3 to 5 years) living in Hong Kong.

**Methods:**

Nominal Group Technique (NGT), a structured, multi-step brainstorming technique, was conducted with two groups of caregivers (mainly parents; *n* = 11) of Hong Kong pre-schoolers in June 2011. Items collected in the NGT sessions and those generated by a panel of experts were used to compile a list of items (n = 22) for a preliminary version of a questionnaire of informal social control. The newly-developed scale was tested with 20 Chinese-speaking parents/caregivers using cognitive interviews (August 2011). The modified scale, including all 22 original items of which a few were slightly reworded, was subsequently administered on two occasions, a week apart, to 61 Chinese parents/caregivers of Hong Kong pre-schoolers in early 2012. The test-retest reliability and internal consistency of the items and scale were examined using intraclass correlation coefficients (ICC), paired t-tests, relative percentages of shifts in responses to items, and Cronbach’s α coefficient.

**Results:**

Thirteen items generated by parents/caregivers and nine items generated by the panel of experts (total 22 items) were included in a first working version of the scale and classified into three subscales: “Personal involvement and general informal supervision”, “Civic engagement for the creation of a better neighbourhood environment” and “Educating and assisting neighbourhood children”. Twenty out of 22 items showed moderate to excellent test-test reliability (ICC range: 0.40-0.81). All three subscales of informal social control showed acceptable levels of internal consistency (Cronbach's α >0.70).

**Conclusions:**

A reliable scale examining PA-related informal social control relevant to Chinese parents/caregivers of pre-schoolers living in Hong Kong was developed. Further studies should examine the factorial validity of the scale, its associations with Chinese children’s PA and its appropriateness for other populations of parents of young children.

## Background

Engagement in physical activity (PA) can benefit children in terms of strengthening their cardiovascular system, enhancing motor and emotional development, and regulating body composition [[1]-[4]]. In 2009, the National Association for Sport and Physical Education (NASPE) in the US proposed PA guidelines for pre-schoolers stating that they should engage in at least 60 minutes of structured and at least 60 minutes of unstructured PA per day, and should not be sedentary for more than 60 minutes at a time except when sleeping [[5]]. Despite these recommendations and the above-mentioned health benefits associated with an active lifestyle, the World Health Organization (WHO) revealed that less than one-third of young people in the world were engaging in sufficient PA for their current and future health benefits [[6]]. Similar findings were reported by Lam, Sit and Cerin [[7]] following an investigation of activity patterns of Hong Kong primary school children.

According to a systematic review, 46% of studies reported that the majority of pre-schoolers did not meet the PA guidelines defined as accumulating at least 60 minutes of moderate-to-vigorous PA (MVPA) per day [[8]]. Using the same guidelines, a recent study in Belgium reported that 48% of 3–6 year olds achieved the recommended levels of PA. The situation in Hong Kong is even worse, with studies reporting that Hong Kong children are among the most inactive in the world [[9],[10]]. For example, Louie and colleagues [[11]] reported that very few pre-schoolers engaged in vigorous PA during 25-minute activity periods in pre-school centres. A recent survey found that Hong Kong children attending pre-school centres accumulated only 45 and 28 minutes of PA when attending whole-day and half-day classes, respectively [[12]]. These findings indicate an urgent need to increase the prevalence of health-enhancing levels of PA in this population.

Determining the factors that promote PA habits adopted in early childhood is of paramount importance given that activity patterns established in this age group are predictive of those later in life[[13]-[15]]. There is empirical evidence that both the physical environment (e.g., availability of PA facilities, aesthetics, and neighbourhood safety) and the social environment (e.g., social support) are potential determinants of PA. Children’s regular engagement in PA may be facilitated by the availability of places for PA in their neighbourhood. The provision of outdoor places for PA appears to be particularly important for young children, who tend to be more active outdoors than indoors [[16],[17]].

When parents choose a place for their young children to play, neighbourhood safety is a major concern. Parental fear of potential harm from strangers (‘stranger danger’), unintentional injury, crime, and traffic hazards may impact on their willingness to allow their children to play outdoors [[18]-[20]]. Of these parental concerns, there is evidence that perceived ‘stranger danger’ and risk of crime are particularly influential in determining parental choices [[18],[19]].

One of the factors that may influence actual and perceived levels of crime and ‘stranger danger’ is the ability of neighbourhoods to maintain effective social control, which is an aspect of collective efficacy [[21]]. Collective efficacy is a form of social capital that includes the willingness of residents to intervene for the good of the community and the linkage of mutual trust within a community [[21]]. Informal social control refers to “the informal mechanism by which residents of a neighbourhood achieve public order”, while the main objective is to construct “safe and orderly environments that are free of predatory crimes”. Parents are likely to regulate the amount of freedom they give their children to play in the local community according to their perception of the level of informal social control. For example, it was found that parents were more likely to allow their children to travel on their own in Jerusalem than in Detroit [[22]]. This difference was attributed to better perceived neighbourhood safety in Jerusalem [[22]]. In another study, children’s MVPA was positively related to parents’ perceived social interaction and cohesion in a particular neighbourhood [[23]]. Parents’ perceptions of informal social control were also found to be positively associated with more walking and cycling to school among children [[22]]. Although no studies have specifically looked at the impact of informal social control on PA-related practices of parents of pre-schoolers and on pre-schoolers’ PA, it is plausible to assume that the positive effects of high informal social control seen in older children would also apply to pre-schoolers.

In order to study the potential effect of informal social control on PA-related parental practices and pre-schooler’s PA, appropriate measures of informal social control are needed. Yet, measures of informal social control relevant to young children’s engagement in PA do not exist [[22]] most likely due to this topic being still understudied in this particular age group. Importantly, such measures need to be tailored to specific geographical locations and cultures, as informal methods of social control vary widely by culture and country [[24]]. Formative studies are needed to identify aspects of informal social control that are relevant to parents and their young children’s PA within specific cultures. Thus, the aim of this study was to develop and assess the reliability of a preliminary version of a scale of informal social control, grounded on formative and qualitative research, appropriate for Chinese parents of pre-schoolers living in high-density urban areas and addressing issues associated with engagement in PA. Physical activity research on Chinese populations is locally and internationally important as China contributes 19% of the world population but the PA patterns and determinants of its urban population are poorly understood.

## Methods

The development of a preliminary version of the scale of PA-related informal social control for Chinese parents of pre-schoolers followed a three-stage process: a formative, qualitative stage involving the conduct of nominal group technique (NGT) sessions; a scale-development stage involving the compilation of items for the scale, cognitive interviews, and scale refinement; and a test-retest reliability stage.

### Formative qualitative stage

#### Participants

In the first stage, a qualitative study was conducted with primary caregivers (an adult who takes primary responsibility of the child – usually, a parent) of 3–5 year-old Hong Kong children to explore their opinions about how residents of their neighbourhood act to make their neighbourhood safe for young children to engage in PA. A convenience sample of participants was recruited from kindergartens, preschool playgroup centres, and Maternal and Child Health Centres (MCHC) of the Department of Health of Hong Kong in 2011. Participants were included if, in the pre-screening questionnaire they identified themselves as the primary caregiver of at least one 3–5 year-old Chinese-speaking child living in Hong Kong. Exclusionary criteria were parents/primary caregivers of children with a disease (e.g. physical disability, severe asthma or cardiovascular diseases) affecting their PA behaviour or cognitive functioning (e.g., Down’s syndromes); and those who were unable to read and write in Chinese. Eligible participants provided informed written consent and were asked to complete a demographic survey prior to participating in the study. Eleven parents/primary caregivers participated in a structured group discussion in June 2011. The characteristics of the participants are reported in Table 1.

**Table 1:** Participants’ characteristics

	**Study component**		
**Characteristics**	**Nominal group technique [n = 11]**	**Cognitive interview [n = 20]**	**Test-retest reliability [n = 61]**
**Gender**			
Male	1	1	5
Female	10	19	56
**Child’s gender**			
Male	4	10	38
Female	7	10	23
**Area socio-economic status**			
Low-middle^a^	5	8	39
Middle-high^b^	6	12	22
**Educational attainment**			
Primary school	2	1	2
Secondary school	6	14	43
Tertiary degree	3	5	16

^a^Average household monthly income: <HKD$20,000; ^b^Average household monthly income: >HKD$20,000.

#### Procedure

The study was approved by the Ethics Committees of the University of Hong Kong and Department of Health and informed consents were obtained from the participants prior to data collection. For this study we used the Nominal Group Technique (NGT), a structured, brainstorming technique used to generate and prioritize responses from a group of participants to a question addressing a specific issue of interest [[25]]. This type of approach has been used in previous scale-development studies involving parents of pre-schoolers [[26]]. Two NGT sessions, one with a sample of 5 participants living in low socio-economic status (SES) (<HK$ 20,000 median monthly household income) and the other in a sample of 6 participants living in a high SES areas (≥HK$ 20,000 median monthly household income), were conducted in MCHCs to identify primary caregivers perceptions of the actions residents would consider to make their neighbourhood safe for children to engage in PA. NGT groups were stratified by SES because there is evidence that informal social control varies by area-level disadvantage [[27]]. Opening statements (e.g., a definition of PA) and questions for the NGT sessions were developed and pre-piloted in a focus group and individual interviews on representatives of the target population to ensure that they could be easily understood by the participants. Two trained facilitators conducted both NGT sessions following strictly defined procedures detailed in a manual.

During the NGT sessions, the parents/caregivers were asked the following question “What sorts of things can people do to make their neighbourhood safe for young children to engage in physical activity?” Participants wrote their responses on a worksheet without discussing them with other participants. Then they were invited one by one in a sequence to nominate one of the responses (items) from their list. This procedure was repeated several times until all ideas were exhausted. Then, they ranked the three listed options that they perceived to be the most relevant. The facilitator collected and tabulated the scores for each item and presented the ranked responses to the group for final group review. The sessions lasted for 60–70 minutes. Responses that were consensually judged by three research team members to be identical or similar were subsequently combined.

### Scale development stage

#### Participants and procedure

The statements collected during the NGT sessions were used to compile a list of items for a working version of a scale of PA-related informal social control relevant to Hong Kong pre-schoolers. The caregiver-generated items (see items denoted by a ^P^ in Table 2) were classified into themes based on the nature of their content by a panel of experts (including the first and second authors), consisting of two psychologists, a nurse, a paediatrician and two public health professionals. The same panel complemented the list of items generated by primary caregivers with additional items based on extant published studies [[21],[22],[24],[28]-[30]], unpublished studies known to the panel members (specifically, the Niños Activos study, [[31]]) and expert knowledge (see items denoted by an ^E^ in Table 2). The working version of the scale consisted of 22 items classified into three categories: “Personal involvement and general informal supervision”; “Civic engagement for the creation of a better neighbourhood environment” and “Educating and assisting neighbourhood children” (Table 2). Each item was rated on a 5-point Likert scale ranging from strongly agree (1) to strongly disagree (5).

**Table 2:** Descriptive statistics and reliability of a scale of physical activity-related informal social control

		**Cronbach’s alpha**				**T1**	**T2**		**Relative frequency (Percentage) of shifts in responses to items**			
**Item**	**People in my neighbourhood…**	**T1**	**T2**	**ICC**	**95% CI**	**Mean (SD)**	**Mean (SD)**	**t (60)**	**0(%)**	**1(%)**	**2(%)**	**3(%)**
**Personal involvement and general informal supervision**		0.74	0.84	0.75	0.61-0.84	2.77(0.54)	2.68(0.57)	1.59				
**1**^**P**^	…supervise the neighbourhood children at all times.			0.57	0.37-0.72	3.18(0.96)	3.03(0.84)	1.38	30(49)	27(44)	4(7)	0(0)
**2**^**P**^	…take turns supervising the neighbourhood children.			0.52	0.32-0.68	3.46(0.89)	3.25(0.77)	2.09*	35(57)	21(34)	5(8)	0(0)
**5**^**E**^	…know and communicate with one another.			0.54	0.33-0.70	2.41(0.84)	2.41(0.76)	0.00	37(61)	20(33)	4(7)	0(0)
**6**^**P**^	…get involved with the neighbourhood children.			0.66	0.50-0.78	2.72(0.84)	2.66(0.87)	0.73	34(56)	26(43)	1(2)	0(0)
**7**^**E**^	…would call the police if something looked strange in our neighbourhood.			0.44	0.21-0.62	2.10(0.63)	2.07(0.66)	0.38	39(64)	20(33)	2(3)	0(0)
**Civic engagement for creation of better neighbourhood environment**		0.90	0.90	0.81	0.70-0.88	2.71(0.67)	2.82(0.63)	−2.32*				
**8**^**E**^	…post “children at play” warning signs when children are out playing.			0.63	0.45-0.76	3.20(1.01)	3.16(0.82)	0.32	32(53)	26(43)	3(5)	0(0)
**10**^**E**^	…organize meetings with the police and other organizations to promote safety.			0.40	0.17-0.59	2.85(0.73)	2.89(0.78)	−0.31	36(59)	21(34)	4(7)	0(0)
**15**^**P**^	…work with the city to ensure that parks are equipped with good facilities for children to play.			0.63	0.44-0.76	2.52(0.92)	2.74(0.85)	−2.20*	27(44)	33(54)	1(2)	0(0)
**16**^**P**^	…work with the city to ensure that parks are well maintained and regularly cleaned for children to play.			0.66	0.42-0.80	2.43(0.90)	2.75(0.79)	−3.93**	29(48)	32(53)	0(0)	0(0)
**17**^**E**^	…work with the city to get more police patrols in our neighbourhood.			0.78	0.66-0.86	2.74(0.79)	2.84(0.76)	−1.52	45(74)	16(26)	0(0)	0(0)
**18**^**E**^	…work with the city to improve street lighting in our neighbourhood.			0.40	0.17-0.59	2.54(0.72)	2.69(0.77)	−1.42	37(61)	20(33)	3(5)	1(2)
**19**^**E**^	…work with the city to reduce traffic speed limits in our neighbourhood.			0.69	0.53-0.80	2.67(0.81)	2.69(0.81)	−0.20	39(64)	21(34)	1(2)	0(0)
**Educating and assisting neighbourhood children**		0.79^#^	0.78^#^	0.61^#^	0.43-0.73^#^	2.13(0.48)^#^	2.17(0.45)^#^	−0.25^#^				
**3**^**P**^	…take children out of a conflict situation.			0.41	0.19-0.60	2.44(0.87)	2.30(0.72)	1.27	33(54)	22(36)	5(8)	1(2)
**4**^**P, D**^	…ensure children have their life-vest or buoy on before playing in the water (sea/lake/ streams/ swimming pool).			0.35	0.10-0.54	2.08(0.71)	2.31(0.79)	−2.23*	37(61)	18(30)	6(10)	0(0)
**9**^**P**^	…will verbally correct a neighbourhood child when his/her parent is not around.			0.34	0.11-0.55	2.38(0.71)	2.36(0.66)	0.16	39(64)	17(28)	5(8)	0(0)
**11**^**E, D**^	…would help a neighbourhood child in need when his/her parent is not around.			0.14	−0.10-0.37	2.07(0.57)	2.28(0.58)	−2.20*	36(59)	21(34)	3(5)	1(2)
**12**^**E**^	…make sure the neighbourhood children do not play in dangerous areas.			0.47	0.25-0.64	2.02(0.67)	2.11(0.69)	−1.10	34(56)	26(43)	1(2)	0(0)
**13**^**P**^	…assist children when they climb on something.			0.56	0.35-0.71	2.26(0.75)	2.28(0.66)	−0.19	37(61)	23(38)	1(2)	0(0)
**14**^**P**^	…discourage children from playing in parks where there are wanderers.			0.42	0.19-0.61	2.31(0.90)	2.21(0.73)	0.86	30(49)	27(44)	3(5)	1(2)
**20**^**P**^	…advise children not to follow strangers.			0.55	0.31-0.71	1.64(0.58)	1.89(0.61)	−3.58**	43(71)	17(28)	1(2)	0(0)
**21**^**P**^	…educate children how to use the facilities correctly to avoid injuries.			0.40	0.17-0.59	2.02(0.62)	2.07(0.60)	−0.57	37(61)	23(38)	1(2)	0(0)
**22**^**P**^	…educate children how to play with other children to avoid conflict.			0.40	0.17-0.59	2.00(0.66)	2.13(0.56)	−1.53	33(54)	28(46)	0(0)	0(0)

Notes. T1 = first assessment; T2 = second assessment; ICC = two-way mixed effects intra-class correlation coefficients; t(60) = t-value (60 degrees of freedom); *p < .01; **p < .001; ^P^ = items generated in Nominal Group Technique sessions by parents/caregivers; ^E^ = items generated by panel of experts; ^D^ = items deleted due to their low ICC and negative impact on Cronbach’s alpha (internal consistency of subscale); ^#^ = computed excluding two deleted items (denoted by ^D^) with low ICC and negatively affecting the internal consistency of the subscale.

The working version of the scale of PA-related informal social control was tested via face-to-face cognitive interviews on a sample of Chinese-speaking 20 primary caregivers of Hong Kong pre-schoolers recruited from and interviewed at kindergartens and MCHCs of the Department of Health in August 2011. Moreover, participants in NGT sessions were contacted and invited to take part in this stage. As a result, six out of the 20 participants took part in both NGT sessions and cognitive interviews. Table 1 shows the distribution of participants who took part in this component of the study by area SES, gender, child’s gender and educational attainment. Eligibility criteria and recruitment procedures for this study component were identical to those of the NGT sessions.

Cognitive interviews are a method developed by Tourangeau [[32]] which consist of the following processes: (1) comprehension of the question; (2) retrieval of relevant information from memory; (3) decision-making; and, (4) responding. Cognitive interviews help the interviewer to understand what a respondent believes a question is asking, including the meaning of words and phrases (e.g., “What does the term ‘neighbourhood’ mean to you?”), how the respondent decides to give a specific answer, as well as whether the respondent can match her/his internally generated answers to the available response categories. The procedure for the cognitive interviews was standardized in the form of scripts that the interviewer had to follow. Interviews were audio-recorded and transcribed verbatim. The information gathered during the cognitive interviews was used to modify the wording of the items (when necessary) and create a final version to be used in the next stage of the scale development (shown in Table 2). All items were retained in the final version of the scale after considering the results from the cognitive interviews, which led to slight rewording of a few items.

### Test-retest reliability stage

A quantitative study was conducted to assess the test-retest reliability of the items included in the newly-developed working version of the scale of PA-related informal social control. Sixty-one participants (none of whom had participated in the qualitative stage of the study) were recruited from kindergartens and MCHCs of the Department of Health in 2012. Participants were included if, in the pre-screening questionnaire, they identified themselves as the parent or primary caregiver of at least one 3–5 year-old Chinese-speaking child living in Hong Kong. Exclusion and inclusion criteria were the same as those for the qualitative components of the study. Eligible participants provided informed written consent. They were asked to complete a demographic survey and the newly-developed self-administered paper-and-pen working version of the PA-related informal social control scale at their convenience, within 7 days of receipt of the survey (first assessment T1). The scale was administered again a week after the completion of the first survey (T2).

The test–retest reliabilities of the items and three a priori determined subscales of PA-related informal social control (“Personal involvement and general informal supervision”; “Civic engagement for the creation of a better neighbourhood environment” and “Educating and assisting neighbourhood children”) were estimated using intraclass correlation coefficients (ICC) based on absolute agreement two-way mixed effects models. The internal consistencies of the three a priori subscales were estimated using Cronbach’s α coefficient. ICCs ≥0.80 were considered excellent, values between 0.60 and 0.79 were considered substantial, values between 0.40 and 0.59 moderate, and ICCs <0.40 were considered poor [[33]]. To establish whether there was a significant change on average scores on the items and subscales from T1 to T2, paired t-tests were performed. The relative percentages of shifts in responses to items were also computed to gain more detailed insight into the test-retest reliability of the individual items. Data were analysed using the SPSS statistical software package version 20.0.0.

## Results

Thirteen items generated during the NGT sessions and nine items generated by the panel of experts were included in a first working version of the scale of PA-related informal social control (Table 2). Five items were classified under the common theme of ‘Personal involvement and general informal supervision”, seven items under “Civic engagement for the creation of a better neighbourhood environment” and 10 items were classified into the theme “Educating and assisting neighbourhood children”.

The test-retest reliability of items representing “Personal involvement and general informal supervision” was moderate to substantial (ICCs: 0.44 – 0.66), with only one item showing a statistically significant but small change in average rating across the two assessments. Also, more than 90% of participants provided answers at T2 that did not differ or differed by only one unit from their responses at T1. The average composite score on the subscale “Personal involvement and general informal supervision” did not significantly change across time and showed substantial test-retest reliability and acceptable levels of internal consistency at both time points (Cronbach’s α >0.70).

The test-retest reliability of items gauging “Civic engagement for the creation of a better neighbourhood environment” was also moderate to substantial (ICCs: 0.40 – 0.78). The average score on two items significantly increased across time, as did the total composite score, but only slightly (Table 2). Most participants did not change their responses or changed them by one unit across time. The test-retest reliability of this subscale was excellent, as was its internal consistency.

Two of the 10 items falling under the category of ‘Educating and assisting neighbourhood children’ had poor test-retest reliability (ICC < 0.40) and showed significant changes in scores across time (items 4 and 11). These items also impacted negatively on the internal consistency of the subscale. Hence, they were left out from the composite score of “Educating and assisting neighbourhood children”. With the exception of the item “People in my neighbourhood will verbally correct a neighbourhood child when his/her parent is not around”, all other items had moderate test-retest reliability (ICCs: 0.40 – 0.56). All items (except the two excluded) positively contributed to the internal consistency of this subscale. Of the retained items, only one showed significant changes in average scores from T1 to T2. The test-retest reliability of this subscale was substantial and internal consistency acceptable at both time points.

Participants tended to report higher levels of agreement to the aspect of “Educating and assisting neighbourhood children” than the other two aspects of informal social control (Table 2; note that lower scores indicate higher levels of perceived social informal control). They agreed the most with the statements “make sure the neighbourhood children do not play in dangerous areas”, “advice children not to follow strangers”, “educate children how to use facilities correctly to avoid injuries” and “educate children how to play with other children to avoid conflict”. Participants disagreed the most with the statements “take turns supervising the neighbourhood children” and “supervise the neighbourhood children at all times” classified under “Personal involvement and informal supervision”, and with the statement “post ‘children at play’ warning signs when children are out playing” classified under “Civic engagement for the creation of a better neighbourhood environment”.

## Discussion

The purpose of this study was to develop and assess the reliability of a preliminary version of a scale of PA-related informal social control relevant to Chinese parents/caregivers of pre-schoolers living in Hong Kong, a high-density urban area in China. To the authors’ knowledge, this is the first study to develop a reliable scale of informal social control specifically relevant to engagement in PA and children of pre-school age. A recent study conducted in a Latino sample developed a scale of informal social control for parents of Latino pre-schoolers which, however, was not specifically focused on aspects of informal social control perceived to affect children’s PA [[34]].

The development of the scale was based on qualitative investigations of parental/caregivers’ and experts’ perceptions of things that residents could do to create safe neighbourhood environments where young children could engage in PA. Most items generated and deemed important by parents/caregivers pertained to things that residents would do to educate and assist neighbourhood young children in developing behaviours that promote personal safety. Although parents enlisted personal involvement, supervision, and civic engagement as relevant aspects of PA-related informal social control, they were not seen to be as important and prevalent as educating and assisting children. All final subscales of informal social control showed acceptable levels of internal consistency.

While previous studies have found that effective informal social control increases the likelihood of parents giving their children more freedom to play in the local community [[22]], no published studies have, to our knowledge, explored parental views about aspects of neighbours’ behaviour that would enhance their perceptions of neighbourhood safety in relation to young children’s engagement in PA. Our findings were consistent with extant studies on social determinants of children’s PA in that parents expressed their concern about potential unintentional injuries and threat from strangers [[19],[35]]. The relevance of risk of unintentional injuries [[19]] was highlighted by parents stating that it was important to ‘assist children when they climb on something’, ‘educate children how to use facilities correctly to avoid injuries’ and ‘educate children how to play with other children to avoid conflicts’. Parental concerns about the harm that strangers could cause to their children while they engage in PA in their neighbourhood [[19],[35]] emerged from their statements that it was important for neighbours to ‘discourage children from playing in parks where there are wanderers’ and ‘advise children not to follow strangers’.

Interestingly, parents participating in the NGT sessions, however, did not generate items describing neighbours’ activities that would specifically address crime and traffic hazard concerns. Such items were introduced by a panel of experts based on prior research acknowledging the importance of crime and traffic safety for engagement in PA [[18],[36]], and of civic engagement and supervision as ways for residents to collectively address such problems [[21]]. Yet, in the quantitative component of this study, participants tended to agree rather than disagree that neighbours would engage in activities that address crime (i.e., would call the police if something looked strange in the neighbourhood; organize meetings with the police and other organizations to promote safety; and work with the city to get more police patrols in the neighbourhood) and traffic hazard concerns (i.e., work with the city to reduce traffic speed limits in our neighbourhood). This indicates that although crime and traffic safety may not be perceived as major safety issues, Hong Kong parents of preschool children, in general, maintain that residents of their neighbourhood would attempt to actively address these problems if needed. The fact that Hong Kong is one of the safest cities in the world with respect to violent crime [[37]] and traffic fatalities [[38]] may explain parents’ tendency to consider threats other than crime and traffic as more important to children’s safety.

As noted earlier, eight out of the 13 items generated during the NGT sessions were categorized as practices related to “Educating and assisting neighbourhood children”. This suggests that Chinese parents of pre-schoolers in Hong Kong maintain that neighbours may substantially contribute to creating safe neighbourhood environments for children by educating them about how to behave properly in the community and by assisting them in learning skills that will enhance the level of personal safety. This finding is in line with the content of extant general, non-PA specific child-centred measures of informal social support that consider assisting children in need and stopping acts of misbehaviour as essential components of the construct of informal social support [[30],[39]].

Another set of findings congruent with previous research regards neighbours’ practices related to personal involvement and general informal supervision of the neighbourhood which were brought up by parents in the qualitative component of the study (i.e., “supervise the neighbourhood children at all times”, “take turns supervising the neighbourhood children” and “know and communicate with one another”). Specifically, previous studies have shown that such behaviours contribute to more positive parental perceptions of environmental safety and, thus, influence parental choices of places where children engage in PA [[40],[41]].

It is noteworthy that, in the qualitative part of this study, parents spontaneously generated only a couple of items describing neighbours’ practices related to civic engagement. These items were less frequently endorsed by parents than items gauging educating and assisting neighbourhood children in developing skills and behaviours contributing to personal safety. This indicates that Hong Kong Chinese adults may, in general, seldom participate in endeavours aimed at neighbourhood-level policy changes, although this type of collective behaviour has been shown to contribute to better community cohesion and interpersonal connections in Western populations [[42]].

The greater emphasis on enhancing neighbourhood safety by shaping the behaviour of young children rather than trying to change the neighbourhood environment through civic engagement observed in this sample may be attributable to the endorsement of a Confucian traditional philosophy typical of Chinese societies [[43]]. An important principle of the political teachings of Confucius is that harmony is achieved if all members of a society loyally perform their role as prescribed in a given hierarchy [[43]]. Confucianism cherishes obedience and adaptation to one’s role in a hierarchy rather than actions aimed to change policies and structures mandated by rulers at the higher levels of the hierarchy. Although Chinese societies have made considerable progress towards democracy and greater civic participation, traditional Confucians values remain to a certain extent embedded in approach to life and choices of action [[44],[45]].

We have developed a preliminary version of a scale of PA-related informal social control appropriate for Chinese parents/caregivers of Hong Kong pre-schoolers. This scale may be also appropriate for use in other urban, developed areas of mainland China and other countries with a high proportion of Chinese residents such as Taiwan, where Confucianism has been found to influence beliefs that shape behaviour patterns and community structure to a greater extent than Buddhism and Taoism[[46]].

### Strengths and limitations

One of the main strengths of our study pertains to the use of qualitative methods to inform the content of a culturally-sensitive scale of informal social control appropriate for Hong Kong parents of pre-school-aged children and focused on children’s PA. Unlike other qualitative methods (e.g., focus groups), the use of the NGT methodology in the qualitative stage of the study provided an opportunity to all participants to make an equal contribution to the content of the scale [[47]]. The use of cognitive interviews to pilot test the newly-developed scale ensured that the items generated were clear and comprehensive. The fact that we recruited participants balanced by child gender and SES ensured that we developed a scale reflecting a wide range of opinions about aspects of PA-related child-centred informal social control in urban Chinese communities.

This study has also several limitations. Our sample consisted primarily of mothers of Hong Kong pre-school children. Given that a recent study on Hong Kong children has shown that informal childcare was linked to higher levels of young children’s obesity [[48]], it would be interesting to examine what other family and household members think about the ways in which neighbourhoods can be made safe for children to play. Another limitation of this study pertains to the fact that it focused on the test-retest reliability and internal consistency of the scale only. Future studies, in larger samples, will need to examine the factorial validity of the scale, i.e., whether the a priori determined groupings of items hypothesised to represent three latent constructs are a sufficiently accurate representation of the empirical patterns of inter-item correlations. The factorial structure of the scale would then also need to be cross-validated with another sample from a different geographical location in China to examine the generalizability of the scale to populations outside Hong Kong. Finally, future work will need to examine the construct validity of the scale in terms of its associations with pre-schoolers’ PA, parental concerns about children’s safety and parental practices related to pre-schoolers’ PA.

## Conclusion

We developed a preliminary version of a scale gauging aspects of neighbourhood PA-related informal social control relevant to Chinese parents/caregivers of pre-schoolers living in Hong Kong, consisting of three subscales assessing “Educating and assisting neighbourhood children”, “Civic engagement for creation of better neighbourhood environment” and “Personal involvement and general informal supervision”. This study suggests that the scale has good test-retest reliability and internal consistency and, thus, can be used to investigate the potential impact of the social neighbourhood environment on Hong Kong pre-schoolers’ PA. This is an issue that remains understudied in Hong Kong as well as other geographical locations. Given that high levels of child-centred informal social control have been associated with lower risk of paediatric overweight/obesity [[49],[50]] and higher levels of PA in children and adolescents [[50]], research into how this construct relates to pre-schoolers’ PA is long overdue. We hope that our newly-developed scale will stimulate research on this topic. Yet, before this can happen, further scale validation work needs to be done. Future studies will need to examine further and in greater detail the metric characteristics of the scale. Specifically, these studies will need to establish the factorial validity of the scale and cross-validate it in other samples of parents. Future studies should also assess the associations of informal social control with children’s PA and other theoretically-related constructs (e.g., parental practices related to pre-schoolers’ PA, perceived neighbourhood crime and traffic safety).

## Competing interest

The authors declare that they have no competing interests.

## Authors’ contributions

YS assisted with data collection, questionnaire development and translation and drafted the manuscript. EC conceived and designed the study, contributed to the questionnaire development, analysed and interpreted the data, drafted and critically revised the manuscript. RM interpreted the data and critically revised the manuscript. All authors approved the final version of the manuscript.
